# Functional significance of macrophages in pancreatic cancer biology

**DOI:** 10.1007/s13277-015-4127-2

**Published:** 2015-09-28

**Authors:** Hai Hu, Feng Jiao, Ting Han, Li-Wei Wang

**Affiliations:** Department of Medical Oncology, Shanghai General Hospital, Shanghai Jiao Tong University School of Medicine, 650 New Songjiang Road, Shanghai, 201620 China

**Keywords:** PDA, Macrophages, Desmoplasia, Angiogenesis, CSCs

## Abstract

Pancreatic ductal adenocarcinoma (PDA) is a lethal disease that is usually diagnosed at late stage with few effective therapies. Despite the rapid progress on the genomics and proteomics of the neoplastic cells, therapies that targeted the pancreatic cancer cells proved to be inefficient, which promoted the researchers to turn their attentions to the microenvironment. Currently, various studies had proposed the microenvironment to be a contributing factor for PDA and pervasive researches showed that macrophages within the malignancy correlate with the malignant phenotype of the disease and were reported to a new therapeutic target. Generally, the pro-tumoral effects of macrophages can be summarized as angiogenesis promotion, immunosuppression, matrix remodeling and so on. Hence, a comprehensive understanding of the biologic behaviors of macrophages and their critical role in PDA development may provide new directions for the managements of the lethal disease. In this review, we will summarize the recent advancements on macrophages as pivotal players in PDA biology and the current knowledge about anti-macrophages as a novel strategy against cancer, with the expectation that more efficient therapies will be developed in the near future.

## Introduction

Pancreatic ductal adenocarcinoma (PDA) is a disastrous disease with an overall 5-year survival rate of less than 5 % and a median survival of less than 6 months [1, 2]. It is estimated that near 40,000 people die of PDA in 2014, rendering it the fourth leading cause of cancer-related death in the USA [1]. Early-stage PDA is asymptomatic, while patients with symptoms were often diagnosed as advanced and metastatic disease with less than 15 % are suitable for surgical resection [3], which offers the only chance of cure. Hence, chemotherapy is the only option for most patients, but gemcitabine, the standard first-line drug for PDA, brings only a modest survival benefits because of chemoresistence [4].

Recently, tremendous progresses had been made on the genomics and proteomics of PDA but no obvious progress had achieved on the prognosis [5, 6], resulting in mounting researchers turned their attentions to the microenvironment, a component that is much more complex than the cancer cells and plays a previously underestimated role in the initiation and progression of the disease [7]. As the most abundant infiltrating leukocytes, pancreatic macrophages (so-called tumor-associated macrophages (TAMs)) were stated to involve in nearly all aspects of PDA biology and were stated as a novel therapeutic target. In the subsequent context, we will summarize the recent knowledge of how TAMs regulate PDA biology and discuss the opportunities to treat PDA by targeting macrophages.

## Pancreatic cancer-associated macrophages

**Fig. 1:**
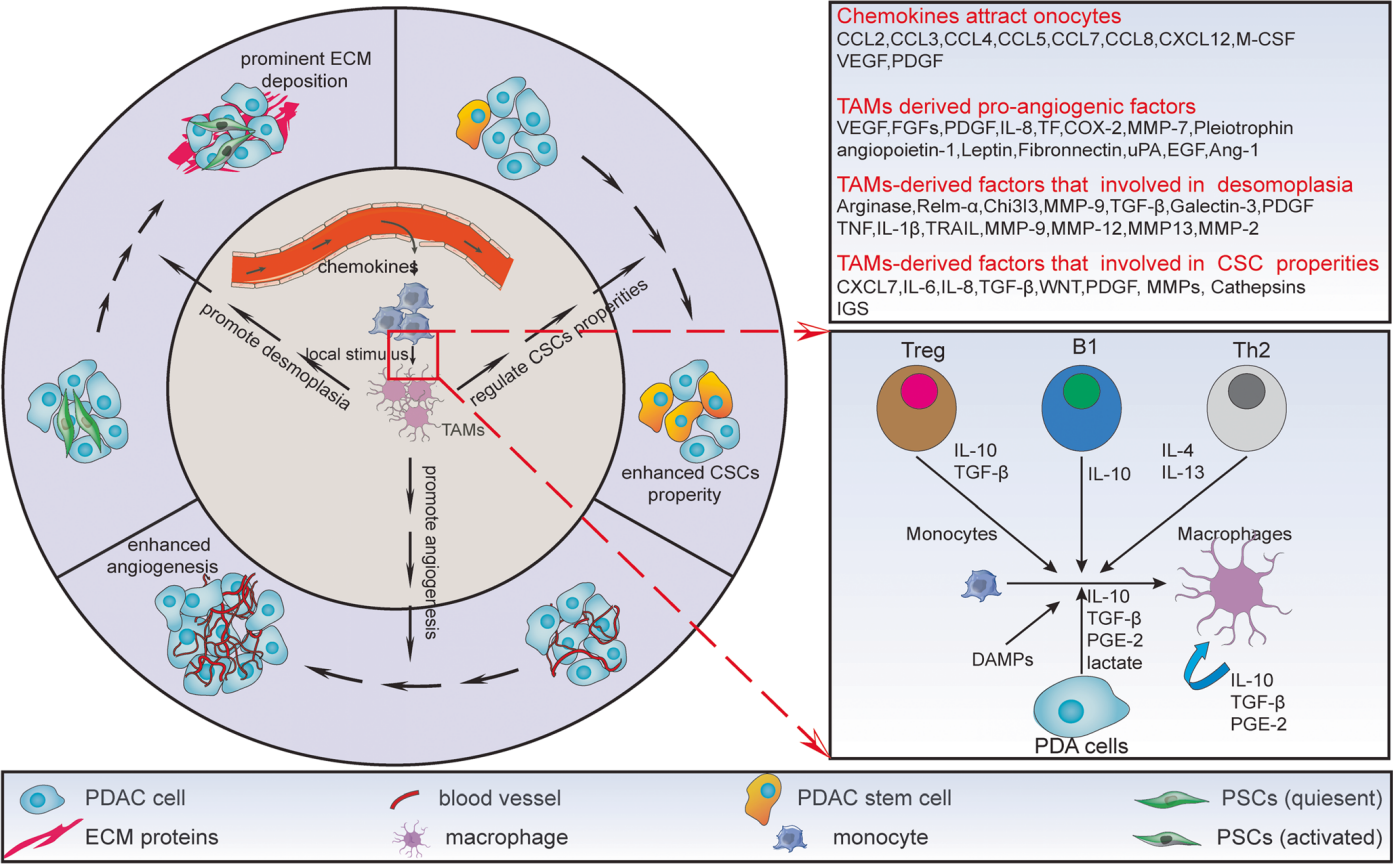
Origin of TAMs and their critical roles in PDA biology. TAMs were derived from monocytes circulating in the peripheral blood. Once recruited to the microenvironment, they underwent differentiation and polarization toward M2 dues to the bioactive molecules within the microenvironment. The transformed M2-like macrophages then exert the pro-tumoral effects directly or indirectly. The *inner circle* describes the recruitment of and polarization of cells; the *outer circle* describes the mechanisms whereby TAMs promote PDA progression. The *upper box* showed the chemokines participated in monocyte recruitment and TAMs-derived factors that regulate angiogenesis, desmoplasia, and CSCs property. The *lower box* showed the detailed factors that promote macrophages polarization. Sources of these factors include the immunocytes, cancer cells, and other stromal cells

Macrophages are leukocytes deriving from the circulating monocytes in the peripheral blood and responsible for homeostasis [8]. In the recent years, line of evidence had tried to clarify how the monocytes were recruited and the mechanism by which they differentiate to macrophages. CSF/CSFR is a commonly accepted signaling that associates with the process [9]. Besides, shattered reports showed that the PK2/PKR and CCL2/CCR2 signaling also involve in leukocytes infiltrating, associating with a poor prognosis [10, 11]. As summarized in Fig. 1, other potent chemoattractants for monocytes include VEGF, PDGF, EMAPII, endothelin, and so on [12]. Recently, M1 and M2 had been described as the functional states of macrophages. Specifically, M1 (so-called classically activated macrophages) are trigged by Th1-related cytokines and bacterial products, express high level of IL-12, and are tumoricidal. By contrast, M2 (so-called alternatively activated macrophages) are activated by Th2-related factors, express high level IL-10, and facilitate tumor progression [13]. The pro-tumoral effects of M2 can be summarized as the promotion of angiogenesis, facilitation of invasion and metastasis, and the protection of the tumor cells from chemotherapy-induced apoptosis [14]. As M1 and M2 were the extremities of polarization, macrophages within the primary tumors tend to be M1-like and/or M2-like.

In fact, the profiles of TAMs are more complex than we can anticipate. First of all, both the phenotypes can be detected in a single tumor exhibiting high heterogeneity, and their effects can be offset by each other. Just as reported, prior studies had identified multiple M1-like and M2-like macrophages in various human malignancies and pathological studies showed that the latter tend to, in most cases, associate with poor outcome. Moreover, TAMs show great plasticity as they could transform reciprocally with the ongoing changing microenvironment. As to PDA, studies also found various TAMs with M2-like in predominance. Consistently, further analysis showed that the M2-like macrophages (characterized by CD68^+^CD163^+^CD204^+^) associated with lymphatic metastasis, distant metastasis, chemoresistence, and hence the survival of the PDA patients [15–21].

**Fig. 2:**
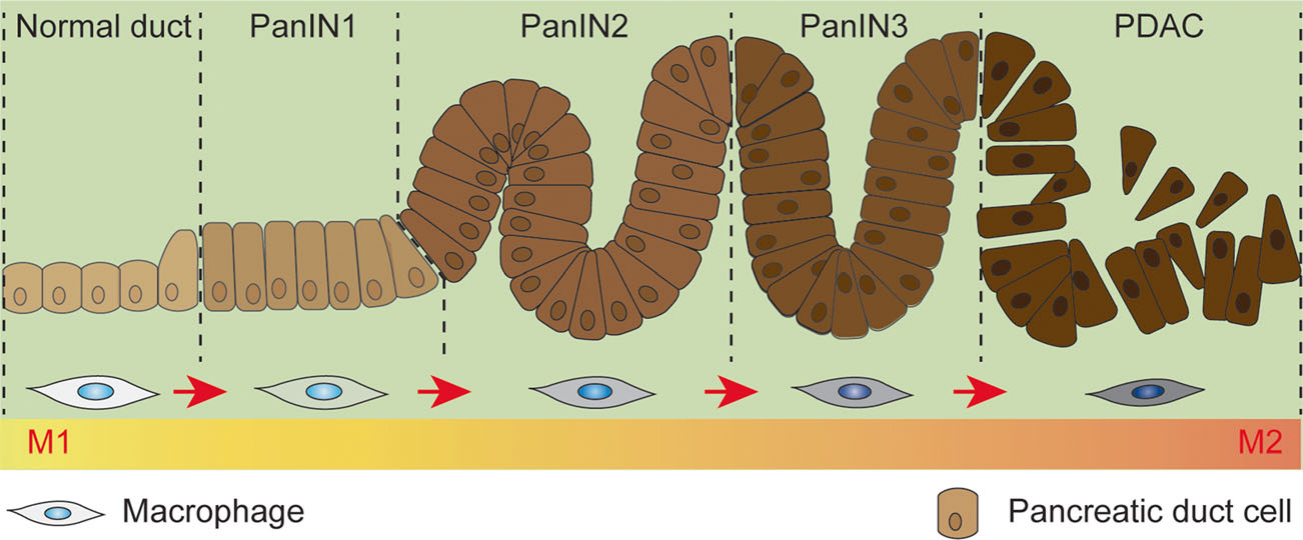
Schematic photograph that describe the initiation and progression of PDA as well as macrophage phenotype changing during the process. Chronic pancreatitis is a risk for PDA. Generally, PDA evolves from intraepithelial neoplasia, accompanied by the macrophage phenotype switching from the anti-inflammation M1-like to pro-tumoral M2-like during the process

Generally, the regional microenvironment, which is featured by dense stroma infiltration, plays a decisive role in recruiting monocytes and modulating macrophage phenotype (Fig. 1). Since different area of a solid tumor exhibits distinct microenvironment, TAMs differ in phenotype as well as function from one region to another. Typically, TAMs in the hypoxia region polarized to M2, while those within the normoxic regions tend to be M1-like [22]. Besides, recent reports suggested that the macrophage phenotype was stage-dependent. For example, the anti-inflammation M1-like macrophages, which usually locate in chronic pancreatitis where tumor occurs, gradually converted to M2-like during tumor initiation and progression [23–25], reflecting the plasticity as well as heterogeneity of TAMs (Fig. 2). As a support, pathological data revealed that M2-like macrophages were more abundant in PDA samples than those diagnosed as chronic pancreatitis [16]. As shown in Fig. 1, numerous factors had been defined as mediators of TAM polarization [14, 26]. Whether they also polarize the pancreatic macrophages toward M2 need further clarified.

## TAMs and desmoplastic reaction

A prominent characteristic of PDA is the formation of a dense stroma termed desmoplastic reaction, which can make up 80 % of the tumor mass in some patients [27]. Initially, the stroma was reported to function as a barrier to limit PDA progression. While, with research continues, many studies re-recognized its pro-tumoral properties and proposed it to be a therapeutic target dues to its critical role in cancer initiation and progression [28]. In defining the source of the stroma, there was report that, in chronic pancreatitis, overactivated PSCs could produce extracellular matrix (ECM) proteins, major component of the dense stroma. Consistently, Apte et al. [29] identified activated PSCs in human PDA samples as the specific source of ECM proteins and the major source of collagen. Functional analysis revealed that elevated PSC activity in human malignancy associated with poor prognosis of the patients [30–32]. Collectively, these studies highlighted the critical role of stroma in PDA progression and proposed that PSCs might play a central role in the formation of the stroma.

PSCs are quiescent with lipid droplets in the cytoplasm. Upon stimulation, they transformed into myofibroblasts with the disappearance of the droplets and were characterized by α-SMA as well as ECM proteins secretion. Some previous studies had identified numerous factors as mediators of PSCs activation and compelling evidence indicated that TAMs might involve in the process via releasing the bioactive mediators. Take PDGF and TGF-β1 for example, compelling evidence had confirmed the fact that TAMs were specific sources of the molecule, and the fact that they could induce the proliferation of PSCs and promote ECM proteins secretion, respectively [33]. In addition, there were some other TAMs-derived pro-fibrotic factors, such as CTGF, CCL17, CCL22, and ROS [34–37]. Correspondingly, there was report that blocking CCR1, a chemokine receptor expressed by TAMs, resulted in reduced TAM infiltration and decreased fibrosis, along with prolonged survival [38]. Of note, activated PSCs can also produce line of effective factors (PDGF, TGF-β1, IL-1, IL-6, COX-2), which, in turn, perpetuate the activation of PSCs [39–42].

Stroma is not just a static mechanical barrier; rather, it consists of a dynamic component, whose turnover was also tightly regulated, mainly by the matrix metalloproteinases (MMPs). MMPs were, in general, secreted by the stromal cells, such as TAMs, and function as regulators of tissue homeostasis by ECM remodeling [43]. Indeed, most prior reports showed that MMPs were overexpressed in most human malignancies, correlating with the malignant phenotype of the disease. For example, studies of skin and cervical cancer showed that TAMs-derived MMP9 could enhance angiogenesis via promoting endothelial cells (ECs) proliferation [44, 45]. As to PDA, Bergers and colleagues found the similar results as they showed that TAMs-derived MMP9 also involved in the stroma turnover by degrading the ECM proteins. More importantly, they also showed in the study that degraded ECM decreased the mechanical stress applied by the stroma on the ECs, leading to enhanced angiogenesis and tumor distant metastasis [46, 47]. Besides, there were other upregulated MMPs members during PDA progression, whether they participate in stroma needs further clarified.

## TAMs and angiogenesis

Angiogenesis could be defined as the growth of new blood vessels from the existing vasculatures, a process usually occurs during the female reproduction cycle and pathological conditions, including cancer. Indeed, in order to grow beyond a certain size, tumors need a dedicated blood supply to provide oxygen and other essential nutrients [48]. However, unlike normal blood vessels, blood vessels from the angiogenic process are dilated with an irregular shape and decreased function and are regulated by the signaling from the cancer cells and stromal cells, including TAMs [49, 50].

As with other solid tumors, PDA also rely on angiogenesis to grow and metastasize [51, 52]. The role of TAMs in angiogenesis was initially recognized due to the correlations between the number of infiltrating TAMs and the vascular density, as ablation of macrophages by targeting integrin α4β1 or myeloid PI3Kγ resulted in decreased blood vessels and reduced tumor burden in mice model of PDA [53, 54]. The study was supported by Tugues [55], who found that conversion of macrophages toward M2-like by depleting histidine-rich glycoprotein (HRG) leading to excessive pro-angiogenic gene expression and increased tumor volume. The mechanism whereby TAMs promote angiogenesis depends on the chemokines, enzymes, and growth factors it secreted, such as VEGF, PDGF, and TGF-β [56]. Besides, in PDA, pancreatic macrophages were known to secret enzymes, such as MMPs and uPAR, to degrade the ECM, and thus modulate the mechanical stress on the endothelial cells (ECs), resulting in enhanced migration along with proliferation and finally enhanced angiogenesis.

Targeting angiogenesis emerged as an effective approach for cancer therapy. A typical case is that the Food and Drug Administration (FDA) approved the usage of bevacizumab in metastatic colorectal cancer patients [6]. Unfortunately, such success does not occur in PDA [57]. Instead, a study by Olive and colleagues [58] proposed “vascular promotion” as a new strategy for PDA managements. They showed in mice model that inhibition of the stroma sonic hedgehog (SHH) pathway could increase vascularization, leading to increased delivery of chemotherapeutic agents to the tumors and greater anti-cancer efficacy. Potential explanations for the contradiction are likely that, the accumulating stroma gradually limits the growth of blood vessels during PDA progression, and thus poor blood perfusion and decreased chemoagent delivery.

Of note, poor blood perfusion often leads to overexpression of hypoxia-inducible factor-1 (HIF-1) in solid tumors, including PDA [59]. As an important transcription factor, HIF-1 was known to regulate the expression of various chemokines that affect angiogenesis. For example, HIF-1-related CXCL12/CXCR4 signaling is potent chemoattractant for ECs [60]. Interestingly, Andrea Casazza also revealed the role of hypoxia in modulating the phenotype of pancreatic TAMs. He showed in mice model that hypoxia could upregulate Nrp-1 expression in TAMs, which functions to recruit the TAMs to the hypoxic region, where they polarized to M2-like and exert anti-immunity response [22].

## TAMs and CSCs

Mounting evidence suggested that malignancies are heterogeneous as their growth and propagation depend on a small subset of cells termed cancer stem cells (CSCs) and defined by their surface marker. As with the normal stem cells, CSCs posses the ability to self-renew and produce differentiated progeny. Prior studies had isolated multiple CSCs from cancers of the prostate, breast, and colorectal [61–63], and accumulating evidence showed that they involved in tumor angiogenesis, distant metastasis, and chemoresistence [64, 65]. As to PDA, Simeone DM showed that CD44^+^CD24^+^ESA^+^ cells isolated from the primary PDA samples posses the ability of self-renew and producing differentiated progeny. The study further identified several aberrantly activated signaling associating with metastasis and self-renew [66]. In addition, Hermann found that CD133^+^ cells in PDA also posses the characteristics of CSCs because as few as 500 of the cells gave birth to tumors that recapitulated the primary tumor when they were injected into immunocompromised mice [67]. This raised the question that whether there is other types of CSCs and cells express all the four markers exhibit stronger CSCs capacity? In the study by Hermann et al. [67], the authors reported an overlap of 14 % between CD44^+^CD24^+^ESA^+^ and CD133^+^ cells but their stemness need to be further clarified.

As mounting evidence support the paradigm that CSCs favor cancer progression via multiple pathways, emerging studies were tried to investigate the formation and maintenance of CSCs property, which were mainly affected by the regional microenvironment. Ding showed in vitro that TAMs could upregulate the stemness and subsequent invasion and migration of breast cancer [68]. Consistently, Hideaki and colleagues [69] reported in mice model that TAMs derived milk-fat globule-epidermal growth factor-VIII (MFG-E8) enhance CSC properties of colon and lung cancer by activating JAK/STAT3 and Sonic Hedgehog (SHH) pathways. Collectively, these researches linked cancer TAMs with CSCs and gave a hint that the pancreatic CSCs might also be regulated by TAMs.

IFN-stimulated gene 15 (ISG15) is a 165-amino acid (17-kDa) protein preferentially secreted by TAMs with a previously underappreciated role in cancer progression [70, 71]. Susana Guerra reported that pancreatic CSCs derived IFN-β could promote TAMs to secret ISG15, which could, in turn, enhance the stemness of PDA both in vitro and in vivo, leading to reinforced capacities of self-renewal and tumorigenicity [72]. In a separate study, depletion of PDA macrophages by inhibiting CSF1R resulted in a significant reduction of cells expressing ALDH (another reported marker of pancreatic CSCs), and improved chemotherapeutic efficacy and anti-tumor responses. Of note, PDA-educated macrophage-derived conditioned media (CM) was sufficient to enhance CSCs properties of murine pancreatic cancer cells in this model [73]. Overall, these data suggested a close correlation between TAMs and CSCs in PDA. However, further study is still needed to determine the detailed crosstalk between them and how these pathways could best be targeted for the patients.

## Targeting macrophage emerges as a novel anti-cancer strategy

**Fig. 3:**
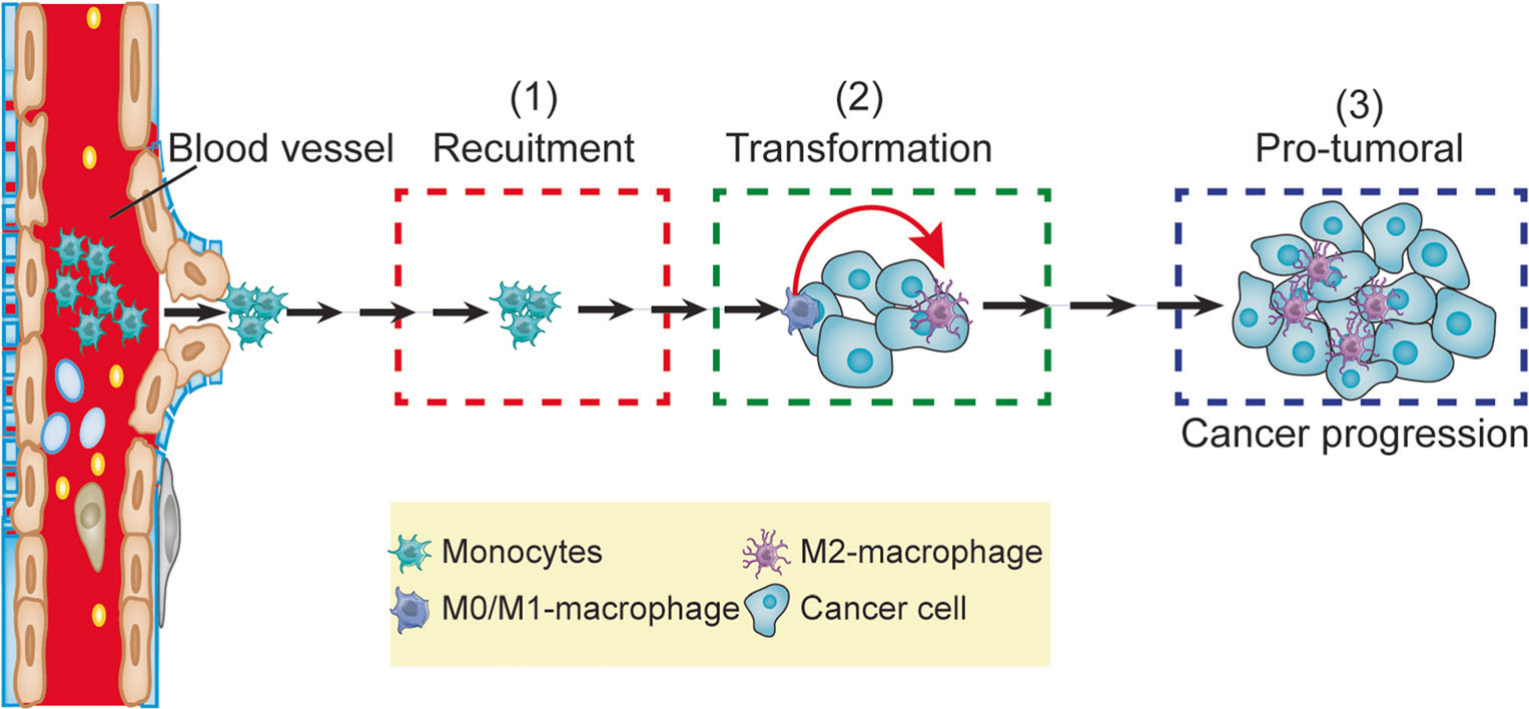
Overview of the recent research focus of macrophages in cancer. The life spans of macrophages include monocyte recruitment, differentiation, polarization, as well as the process they exert their function. The recent studies were mainly tried to clarify how the monocytes were recruited to the microenvironment, how the macrophages were polarized, and how the transformed cells exert their effects

Considering the functional significance of macrophages in cancer initiation and progression, mounting studies had conducted to evaluate the efficiency of anti-macrophage as a novel strategy against cancer. Generally, the life span of macrophage includes monocytes recruitment, differentiation, polarization, and the pro-tumoral process (Fig. 3). Accordingly, the anti-macrophage strategies include the inhibition of the monocytes recruitment as well as transformations, and the ablation the macrophages directly. Some excellent studies of such strategies are listed in Table 1. Collectively, these studies proposed anti-macrophages to be a novel approach for cancer management.

**Table 1:** Excellent studies that target macrophages as a new strategies against cancer

Excellent studies targeting monocyte recruitment		
Signaling	Comments	Refs
PK2/PKR	Gemcitabine in combine with PKRA1, a small molecule PK2 antagonist, could prolong the survival of pancreatic xenograft models via blocking myeloid cell migration	[11]
CCL2/CCR2	In orthotropic model of murine PDA, CCR2 inhibition depletes monocytes and macrophages within the primary tumor, resulting in decreased tumor growth	[10]
CSF/CSFR	CSF-1R inhibition with RG7155, a monoclonal antibody, strongly reduces pro-tumoral macrophages and enhances the immunity, leading to striking clinical objective responses in diffuse-type giant cell tumor patients	[9]
GM-CSF/GM-CSFR	Kras mutation in pancreatic ductal cells triggers the production of GM-CSF, which, in turn, promotes the recruitment of monocytes, leading the accumulation of immunosuppressive macrophages and cancer progression	[74]
Excellent studies targeting macrophage polarization		
Cox-2	Cox-2 and its products involved extensively in M2 polarization and hence cancer progression	[75, 76]
Lactate	Lactic acid produced by tumor cells functions in M2 polarization, a process mediated by hypoxia-inducible factor 1, and favors tumor growth via lactate-induced arginase 1 by macrophages	[77]
SHIP	The src homology-2 domain containing inositol polyphosphate 5-phosphatase (SHIP) functions to repress M2 skewing. Peritoneal macrophages from SHIP^−/−^ mice promote tumor growth	[78]
Legumain	Vaccine against M2-associated molecule legumain induced a robust CD8^+^T cell response against TAMs, resulting in the suppression of tumor growth	[79]
Excellent studies that targeting macrophages survival		
Yondelis	Yondelis, a antitumor agent that inhibits NF-Y, has a unique toxicity for TAMs, leading to decreased macrophages in the microenvironment	[80]
Clodronate	Treatment with clodronate encapsulated in liposomes (clodrolip) depleted macrophages in xenograft model of rhabdomyosarcoma, resulting in decreased tumor growth	[20]

## Concluding remarks

As evidence continually mounts to support that TAMs dictate the biologic behavior of PDA, it is undoubted that much work was needed to understand the molecular machinery whereby TAMs polarize and transform, and much is yet to be learned about the crosstalk between TAMs and other resident cells. Such studies are likely to yield important insights into PDA biology, which may ultimately improve therapeutic approaches and outcomes for the patients.
